# MXene-Based Flexible Paper Chip for Glucose Detection in Sweat in Low-Temperature Environments

**DOI:** 10.3390/s25144273

**Published:** 2025-07-09

**Authors:** Yandong Yang, Yajun Zhu, Yifei Wu, Fan Chang, Xu Zhu, Xinyue Zhang, Ning Ma, Yushu Wang, Alaa S. Abd-El-Aziz

**Affiliations:** 1 College of Material Science and Chemical Engineering, Harbin Engineering University, Harbin 150001, China; yangyd@hrbeu.edu.cn (Y.Y.); zhuyajun@hrbeu.edu.cn (Y.Z.); wuyifei@hrbeu.edu.cn (Y.W.); cf345@hrbeu.edu.cn (F.C.); lf218029@hrbeu.edu.cn (X.Z.); xinyue@hrbeu.edu.cn (X.Z.); nma@hrbeu.edu.cn (N.M.); 2 Qingdao Innovation and Development Center, Harbin Engineering University, Qingdao 266000, China; 3 Shanghai Composite Technology Co., Ltd., Shanghai 201112, China

**Keywords:** MXene-based sensor, photothermal, glucose detection, diabetes, glucose oxidase

## Abstract

In enzymatic reaction glucose detection chips, the enzyme can easily dislodge from the electrode, which harms both the chip and test stability. Additionally, enzyme activity significantly decreases at low temperatures. Consequently, immobilizing the enzyme at the appropriate substrate and ambient temperature is a critical step for improving the chip. To address this issue, an electrochemical detection chip was modified using the nanomaterial MXene, known for its large specific surface area, excellent adsorption, good dispersion, and high conductivity. Meanwhile, AgNO_3_ solution was added to the Ti_3_C_2_T_x_ MXene nanosheet solution, and the AgNP@MXene material was prepared by heating in a water bath. This process further enhances photothermal conversion efficiency due to the localized surface plasmon resonance effect of silver nanoparticles and MXene. This MXene-based photothermally enhanced paper chip exhibits outstanding photothermal conversion performance and sensitive photoelectrochemical responsiveness, along with good cycling stability. Moreover, improved glucose detection sensitivity at low winter temperatures has been achieved, and the ambient temperature range of the paper chip has been expanded to 25–37 °C.

## 1. Introduction

Diabetes has been shown to have a significant adverse impact on human health [1]. The number of people living with diabetes has increased dramatically worldwide in recent years, with approximately 537 million individuals currently affected by the condition. Projections indicate that this number will rise to 783 million by 2045 [2]. It is clear that regular blood glucose monitoring in patients experiencing hyperglycemia, when paired with the timely implementation of therapeutic measures, can help reduce the risk of complications. Consequently, the precise measurement of glucose concentration in human blood is crucial [3,4,5]. Currently, the most common method of blood glucose testing involves invasive blood sampling, which can result in infection and discomfort for the individual. Therefore, a variety of non-invasive detection methods have been developed [6,7,8,9,10,11]. Due to the strong correlation between glucose levels in blood and sweat, several non-invasive sweat-based glucose tests have been created to replace the use of invasive blood glucose sensors [12,13,14,15]. Based on the working principles of glucose detection chips, they can be classified into enzyme-based glucose sensors and non-enzymatic glucose sensors. In enzyme-based glucose detection, glucose oxidation occurs in the presence of enzymes and reaction mediators. Glucose detection relies on the oxidation reaction of glucose, which produces gluconic acid. Non-enzymatic glucose detection is achieved without enzymes, offering enhanced stability but typically exhibiting lower selectivity and sensitivity. In contrast, enzyme-based sensors leverage highly specific catalytic reactions for glucose oxidation, delivering superior selectivity and sensitivity [16,17,18]. However, glucose oxidase (GOx), a type of biological macromolecule, shows significant sensitivity to environmental pH and temperature. The reaction rate of GOx has an exponential relationship with temperature; for instance, an increase of 10 °C in temperature results in a twofold increase in the enzyme’s reaction rate. The optimal temperature range for GOx is 25–40 °C, and its activity significantly diminishes at temperatures below 25 °C, which is highly detrimental to the detection process [19,20].

**Scheme 1:**
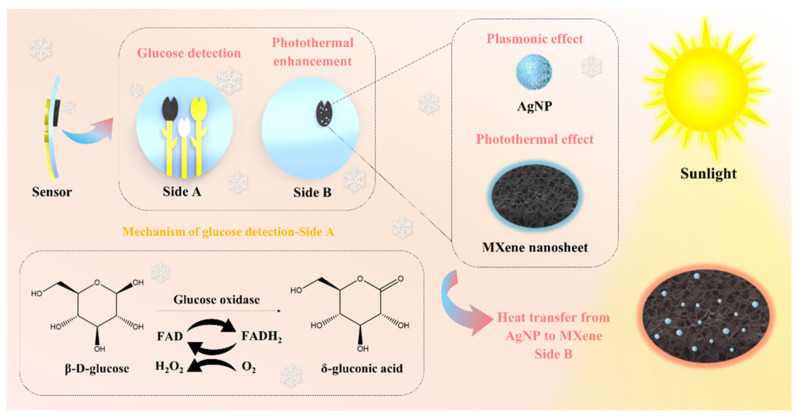
Schematic diagram of AgNP@MXene-based paper chips undergoing photothermal conversion under light illumination for enhanced glucose detection.

In this work, we developed a photothermal paper chip electrochemical sensor for glucose detection in sweat at low temperatures. MXene is a new class of two-dimensional materials consisting of layered compounds of transition metal and carbon or nitrogen. The general chemical formula is usually written M_n+1_X_n_T_x_, where M represents the transition metal, X represents carbon or nitrogen, and T_x_ is a surface-modified functional group. MXene is obtained by selectively etching the aluminum layer in MAX-phase materials (e.g., Ti_3_AlC_2_) and exfoliating it. MXene has a unique lamellar structure and exhibits high electrical conductivity and mechanical strength. The abundance of functional groups on its surface also gives it good hydrophilicity and tunable chemical properties [21,22,23,24,25,26]. The use of this material has been extensively applied in the field of wearable sensor development and electrocatalytic transformations [27,28,29,30,31]. The development of a double-sided, three-electrode, paper-based chip for the detection of glucose in sweat at low temperatures utilizing an enzymatic reaction mechanism was the objective of this study. As demonstrated in Scheme 1, where the A-side provides glucose detection capability, the ink containing monolayer MXene and GOx is utilized as the working electrode, and the AgNP@MXene ink on the B-side provides the photothermal conversion capability. In the context of glucose electrochemical detection, FAD (oxidized flavin adenine dinucleotide) constitutes the active central prosthetic group of GOx. Upon the oxidation of glucose, the FAD molecule accepts two electrons and two protons, undergoing a reduction to form the FADH_2_ compound. The incorporation of monolithic layers of MXene and Ag nanoparticles not only enhanced material stability but also increased the specific surface area, improving the electrical conductivity and electrochemical properties. The photothermal enhancement effect is achieved through the dual LSPR effect of Ag nanoparticles and MXene, significantly improving detection sensitivity in low-temperature environments and extending the paper chip’s usable temperature range to 25–37 °C [32]. It is anticipated that this advancement will enable applications in the field of wearable flexible electronics in the future.

## 2. Experimental Section

### 2.1. Materials

Hydrochloric acid (HCl, 99.7%) and silver nitrate (AgNO_3_, 99.7%) were purchased from Sinopharm Chemical Reagent Co., Ltd. (Shanghai, China). Lithium fluoride (LiF, 99.7%), Nafion117 solution (C_14_F_28_O_6_S_4_, 5%), anhydrous sodium dihydrogen phosphate (NaH_2_PO_4_, 99.7%), and β-D-glucose (C_6_H_12_O_6_, 85%) were purchased from Shanghai McLean Biochemistry Technology Co., Ltd. (Shanghai, China). Ti_3_AlC_2_ MAX (200 mesh, 98%) was purchased from Jilin Eleven Technology Co., Ltd. (Jilin, China). Calcium hydroxide (Ca(OH)_2_, 99.7%) was purchased from Tianjin Tianli Chemical Reagent Co., Ltd. (Tianjin, China). Silver/silver chloride conductive silver paste (Ag/AgCl, 80:20) was purchased from Shanren New Material Technology Co., Ltd. (Jiaxing, China). Anhydrous disodium hydrogen phosphate (Na_2_HPO_4_, 99.7%) was purchased from Beijing InnoChem Science & Technology Co., Ltd. (Beijing, China). Glucose oxidase (C_6_H_12_O_6_, ≥10 KU) was purchased from Sigma Aldrich Trading Co., Ltd. (Shanghai, China). Anhydrous ethanol (CH_3_CH_2_OH, 99.7%) was purchased from Tianjin Yongda Chemical Reagent Co., Ltd. (Tianjin, China).

### 2.2. Preparation of Monolayer MXene

First, HF was prepared by adding 1.6 g of LiF to 9 M 20 mL of HCl and stirring for 20 min. Then, 1.0 g Ti_3_AlC_2_ powder was added slowly at 35 °C for 24 h. Next, the mixture was washed with deionized water and centrifuged at 5000 r/min for 5 min, until the supernatant was close to neutral and the clay-like deposit at the bottom was multilayered Ti_3_C_2_T_x_. Then, 30 mL of deionized water was added to the obtained multilayered Ti_3_C_2_T_x_, injected into Ar_2_ for 10 min, and shocked at 2000 r/min for 3 h. Subsequently, it was kept at 4 °C for 8 h. After, it was immediately placed in a refrigerator freezer (−20 °C) until completely frozen. The freezing–thawing process was repeated for 10 cycles. Finally, the mixture solution was centrifuged at 2500 r/min for 1 h, and the supernatant obtained was the monolayer Ti_3_C_2_T_x_ MXene solution.

### 2.3. Preparation of MXene Ink

The Nafion117 solution and monolayer Ti_3_C_2_T_x_ MXene solution were mixed at a volume ratio of 1:10 and ultrasonicated for 5 min. A quantity of 4 mg of GOx was amalgamated with 1 mL of the mixed solution, and ultrasonicated for 5 min to produce the MXene ink. An AgNO_3_ solution was prepared by taking 3.15 mg of AgNO_3_ powder, dissolving it in 10 mL of deionized water, and then adding it to a monolayer Ti_3_C_2_T_x_ MXene solution. The resulting solution was then heated in a water bath at 40 °C for 30 min, and subsequently subjected to centrifugation at 10,000 rpm for 10 min, which was repeated on two occasions to obtain AgNP@MXene. AgNP@MXene ink was obtained by adding deionized water to AgNP@MXene.

### 2.4. Preparation of Photothermally Enhanced Paper Chips

A laser-engraved skeletonized aluminum plate was used as a mask plate, and a Ti layer was magnetically sputtered on a commercial mixed cellulose (MCE) film for 5 s, and a Au layer for 75 s. MXene ink was applied to the upper left corner of the paper chip as the working electrode, Ag/AgCl (80:20) conductive silver paste was applied to the middle area as the reference electrode, and the magnetron-sputtered Au layer in the upper right corner of the paper chip was used as the auxiliary electrode. On the reverse of the working area coated with MXene ink, AgNP@MXene solution was coated with a brush, and the photothermally enhanced chip preparation was completed.

### 2.5. Electrochemical Characterization of Photothermally Enhanced Paper Chips

An aqueous phosphate-buffered saline (PBS) solution with a concentration of 0.1 mol/L and a pH of 7.4 was first configured. The electrochemical sensing test used the above PBS solution as the electrolyte, which was used to simulate the human sweat environment. The three-electrode region of the paper chip was immersed in the PBS solution or the PBS solution containing glucose for the test. The scanning speed for the cyclic voltametric curve test of the paper chip was set to 100 mV/s, 150 mV/s, 200 mV/s, 250 mV/s, and 300 mV/s, respectively, and the scanning interval was set to −1.2–1 V, with a sampling interval of 0.001 V. The working potential for the time–current curve test of the paper chip was set to 0.75 V, and the sampling interval was set to 0.1 s.

### 2.6. Photothermal Sensing Testing of Photothermally Enhanced Paper Chips

An optical power meter was used to determine the optical power at the focal point of the light beam, and the paper chip was placed at the focal point of the light beam with the A-side facing up. Then, the infrared thermal imager and contact thermometer were used to record the temperature change in the A-side and B-side of the paper chip under different optical powers, respectively. The infrared thermal imager was fixed with a tripod to ensure that the temperature measurement frame of the infrared thermal imager was consistent with the center of the paper chip.

### 2.7. Material Characterization

A scanning electron microscope (SEM, IT300LV, JEOL, Tokyo, Japan), a transmission electron microscope (TEM, Tenai G2 F30, FEI, OR, USA), and an Atomic Force Microscope (AFM, Dension Icon, Bruker, Karlsruhe, Germany) were adopted to observe the microscopic morphology of Ti_3_AlC_2_, MXene, or Ag@MXene. The chemical structure and elemental composition of the materials were further analyzed with an X-ray diffractometer (XRD, TTR-Ⅲ, Rigaku, Tokyo, Japan), an X-ray photoelectron spectrometer (XPS, 250Xi, Thermo, MA, USA), and an Ultraviolet–Visible Spectroscope (UV–Vis, SP−1702, Shanghai spectrum, Shanghai, China). A Contact Angle Measuring Instrument (DSA25, KRUSS, Hamburg, Germany) was adopted to measure material surface wettability. A Four Probe Resistivity Tester (HPS2663, HELPASS, Changzhou, China) was adopted to measure surface conductivity. An infrared thermal imager (Tix560, Fluke, WA, USA) and a contact thermometer (YET610, YOWEXA, Shenzhen, China) were adopted to measure the surface temperature. Electrochemical procedures were performed on an electrochemical workstation (CHI660, Shanghai Chenhua, Shanghai, China).

## 3. Results and Discussion

### 3.1. Characterization of AgNP@MXene

**Figure 1:**
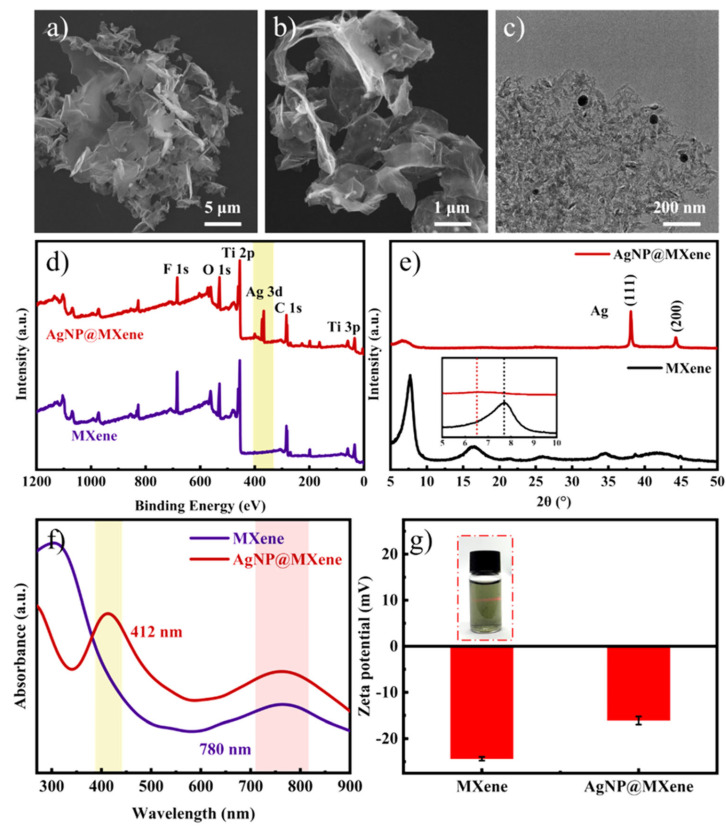
(**a**) SEM image of multi-layer MXene. (**b**) SEM image of AgNP@MXene. (**c**) TEM image of AgNP@MXene. (**d**) XPS survey spectra of MXene and AgNP@MXene. (**e**) XRD patterns of MXene and AgNP@MXene. (**f**) UV–Vis absorption spectra of MXene and AgNP@MXene. (**g**) Zeta potential of MXene and AgNP@MXene.

Due to the co-intercalation of Li^+^ and water molecules, the MXene lamellae were completely opened; clear MXene lamellae can be seen, as shown in Figure 1a, and the lamellar connection changes from dense to loose. Figure 1b shows the SEM image of AgNP@MXene. It can be seen that many smaller-sized dot particles are uniformly distributed on the surface of the MXene lamellae, and almost no aggregation occurs between the dots, which indicates that the Ag nanoparticles are more dispersed on MXene, and AgNP@MXene hybrids are formed. Further observation via transmission electron microscopy is shown in Figure 1c, in which the dark-colored particles with nearly spherical shapes are Ag nanoparticles, and the light-colored lamellar material is the MXene nanosheets, and it can be observed that about twenty Ag nanoparticles are distributed on the MXene lamellae in a range of sizes from 10 to 50 nm, and are dispersed with almost no aggregation. According to the energy dispersive XRD of Ti, C, O, F, and Ag elements shown in Figure S1, it can be seen that Ti, C, O, and F elements are uniformly distributed while Ag elements are dispersed, which further verifies that Ag nanoparticles are uniformly dispersed on the surface of MXene nanosheets, and that the AgNPs are successfully anchored on the MXene nanosheets.

The high-resolution TEM image of AgNP@MXene hybrids is shown in Figure S2, where the dark-colored spheres are magnified Ag nanoparticles. The inset shows the selected electron diffraction of the high-resolution image of the dark-colored spherical region, the pattern of which consists of very neatly aligned parallelogram spots, which is a typical electron diffraction pattern of single crystals, verifying the single-crystal structure of the Ag particles. The local magnification of the light-colored material at the edge in the white box shows that the lattice spacing of the MXene nanosheets is about 0.244 nm. The local magnification of the dark-colored spherical material in the red box shows that the lattice spacing of the AgNP is about 0.250 nm, which agrees with the (111) facet of the Ag crystals. AgNP@MXene was further characterized by AFM. As shown in Figure S3, the heights of the silver particles were 13.15 nm, 7.72 nm, and 10 nm in the direction of Line 1, and 7.08 nm and 10.45 nm in the direction of Line 2. It was demonstrated that AgNP@MXene hybrids contained Ag nanoparticles with a particle size of about 10 nm.

The AgNP@MXene structure was further characterized. As shown in the X-ray diffraction spectra of Figure 1d, for MXene and AgNP@MXene, the two diffraction peaks at 2θ of 38.09° and 44.29° belong to the (111) and (200) crystal planes of the face-centered cubic structure of metallic silver, respectively, which confirms the formation and crystalline nature of AgNPs in the AgNP@MXene hybrids. The characteristic peaks of MXene shifted from 7.6° to 6.7°, indicating that the spacing between the MXene nanosheets widened, further demonstrating that the AgNPs are anchored to the MXene nanosheets. The surface chemistry of AgNP@MXene was further characterized by X-ray photoelectron spectroscopy. As shown in Figure 1e, the characteristic peak was observed at around 367 eV, which is related to the characteristic peak of Ag 3d. Figure S4 shows the XPS spectra of the Ag 3d nuclear energy level of AgNP@MXene, in which two characteristic peaks are located at 367.36 and 373.36 eV, corresponding to the binding energies of Ag 3d_5/2_ and Ag 3d_3/2_, respectively, and the difference in the binding energy between the two peaks is 6.0 eV. The spin energy separation between the three-dimensional double states of Ag is also close to 6.0 eV, suggesting the self-reduction of silver. It is demonstrated that Ag nanoparticles were successfully introduced onto the MXene surface for the preparation of AgNP@MXene hybrids.

The UV–Vis–NIR spectra of MXene and AgNP@MXene hybrids are displayed in Figure 1f. A pronounced absorption peak centered at 412 nm was evident in the AgNP@MXene hybrid, attributable to the surface plasmon resonance peaks of the Ag nanoparticles. This finding indicates that AgNP@MXene exhibits an augmented absorption range of visible light in comparison with pure MXene, thereby facilitating enhanced photothermal conversion efficiency. The zeta potential was further employed to demonstrate the formation of hybrids. As demonstrated in Figure 1g, the surface charge of MXene is estimated to be approximately −24.37 eV, while AgNP@MXene exhibits a charge of −16.1 eV. This reduction is attributed to the coalescence of MXene and Ag nanoparticles. The inset provides a photograph of MXene dispersed under the Tyndall effect.

### 3.2. Characterization and Electrochemical Properties of AgNP@MXene-based Paper Chips

The surface chemistry of Ti_3_C_2_T_x_ is crucial for its properties and applications. XPS was used to examine the composition of layered Ti_3_C_2_T_x_, determine electron binding energies, and analyze its surface chemical properties and composition. The results are shown in Figure S5. Figure S5a presents the full XPS spectrum of layered Ti_3_C_2_T_x_. The F 1s peak appears due to the introduction of F-terminating groups during the etching process. Figure S5b–d show the high-resolution spectra of Ti 2p, C 1s, and O 1s, respectively, fitted with Gaussian–Lorentzian curves. The Ti 2p high-resolution spectrum reveals four fitted peaks at 454.9, 455.8, 457.0, and 459.0 eV, corresponding to Ti-C, Ti-O, Ti_x_O_y_, and Ti-F bonds. The higher content of Ti-O bonds indicates abundant oxygen-containing functional groups on the surface. The C 1s spectrum consists of four peaks at 281.8, 284.5, 285.4, and 288.7 eV, assigned to C-Ti, C-C, C-O, and C-F bonds. The O 1s spectrum displays four peaks at 529.6, 530.6, 531.8, and 532.9 eV, attributed to TiO_2_, C-Ti-O_x_, C-Ti-(OH)_x_, and O-Ti-(OH)_x_ groups. These results confirm that chemical etching successfully introduced abundant terminal groups (e.g., -F, -O, -OH) onto the Ti_3_C_2_T_x_ surface, leading to improved hydrophilicity of the Ti_3_C_2_T_x_ nanosheets.

**Figure 2:**
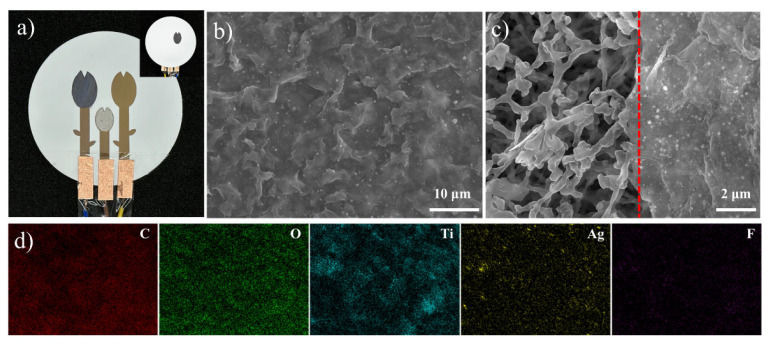

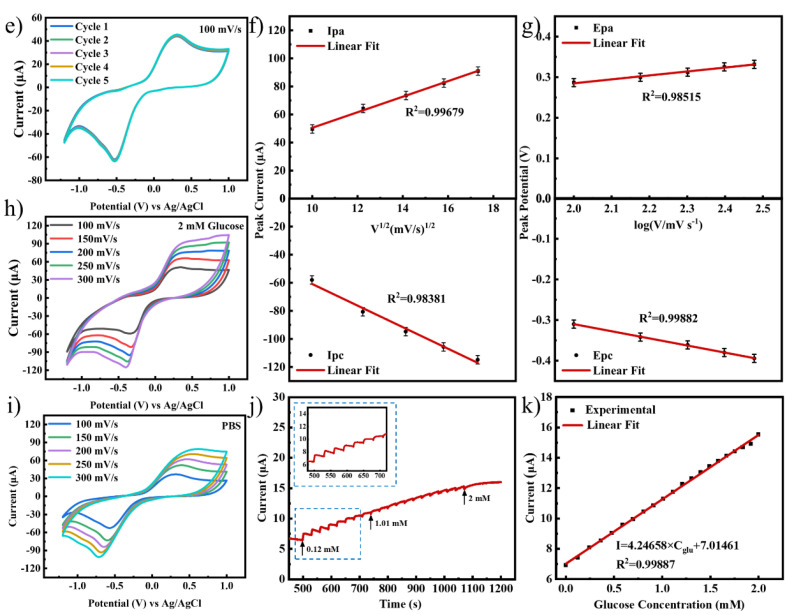
(**a**) Photograph of paper chips. (**b**) SEM image of AgNP@MXene writing marks on MCE membrane surface. (**c**) SEM images of AgNP@MXene writing trace demarcation lines on the MCE membrane surface. (**d**) EDX elemental mapping images of AgNP@MXene (C, O, Ti, Ag, and F). (**e**) Cyclic voltammetry curves of paper chips at a scanning speed of 100 mV/s. (**f**) Relationship between the anode and cathode peak current values and the square root of the scanning speed. (**g**) Relationship between the anode and cathode peak potential values and logV. (**h**) Cyclic voltametric curves of paper chips in 2 mM glucose solution. (**i**) Cyclic voltametric curves of paper chips in PBS solution. (**j**) Time–current curve of glucose response test (inset is partially enlarged). (**k**) The relationship between the current value and the glucose concentration.

Further FTIR spectroscopic analysis of the sample was performed, as shown in Figure S6. An absorption band was observed at approximately 3433.7 cm^−1^, indicative of the prevalence of hydrophilic groups on the surface of MXene. The SEM image of MXene writing on the A-side of the paper chip is shown in Figure S7. This figure demonstrates that the Ti_3_C_2_T_x_ nanosheets have been successfully attached to the MCE substrate and that the layer is dispersed more uniformly. This indicates that the working electrode surface of the paper chip was completely written, forming a continuous writing trace and achieving a continuous conductive pathway. The optical photomicrograph of the photothermally enhanced glucose detection paper chip is shown in Figure 2a. AgNP@MXene was deposited onto the B-side of the paper chip using a brush, and the SEM images of its writing traces at different magnifications are shown in Figure 2b. The MXene lamellae exhibited complete coverage of the MCE substrate, with the Ag nanoparticles being uniformly dispersed on the structure of the MXene lamellae. This suggests that the AgNP@MXene was completely written onto the surface of the MCE membrane, resulting in uniform coverage. As illustrated in Figure 2d, the EDS elemental mapping reveals the presence of the elements carbon, oxygen, titanium, silver, and fluorine. Figure 2c presents the SEM image of AgNP@MXene on the B-side of the paper chip, where the boundary of the writing trace is located. This method allows for the coverage of the underlying fibrous MCE membrane, and the writing boundary is visible.

To further explore the electrochemical stability of the new paper chip, Figure 2e shows the CV curves of the paper chip in a solution of PBS. It can be observed that for a given paper chip, the five circles of CV curves essentially overlap, indicating that the paper chips exhibit adequate stability. The electrode reversibility of paper chips was tested and characterized using cyclic voltammetry. The detection area distribution of the paper chip was placed in a PBS solution, as illustrated in Figure 2f. The oxidation peak potential exhibited a positive shift with an increase in scanning speed, while the anodic peak current value demonstrated a corresponding increase. Conversely, the reduction peak potential shifted negatively with an increase in scanning speed, increasing the cathodic peak current value. The effect of scanning speed (V) on the current and potential was also explored, as demonstrated in Figure 2h. As the scanning rate was increased from 100 mV/s to 300 mV/s, the redox current response value increased gradually. By comparing the cyclic voltammograms in 2 mM glucose aqueous solution and PBS solution, as shown in Figure 2h,i, it can be seen that the electrode undergoes redox reactions regardless of the presence of glucose [33]. In the absence of glucose, GOx-FAD undergoes a reduction reaction at the electrodes to form GOx-FADH_2_, and GOx-FADH_2_ is re-oxidized by oxygen, producing H_2_O_2_ in this process. Therefore, the electrochemical mechanism can be further determined based on the presence or absence of glucose oxidase in the electrode, as shown in Figure S8. It is evident that in the absence of glucose oxidase, the cyclic voltammograms do not exhibit a redox peak. Therefore, it can be determined that the redox reaction comes from glucose oxidase. The CV curve demonstrates the electrochemical response of the MXene-based glucose sensor. The primary oxidation peak at approximately 0.25 V corresponds to the oxidation of H_2_O_2_ generated from the GOx-catalyzed reaction (H_2_O_2_ → O_2_ + 2H^+^ + 2e^−^). The reduction peak around −0.4 V should be the reduction of FAD to FADH_2_ (Scheme 1) caused by accepting the electrons generated from glucose dehydrogenation (FAD + 2H^+^ + 2e- → FADH_2_) [34]. A weak oxidation peak observed at higher potentials (~0.8 V) may originate from the oxidation of functional groups on the MXene surface [35]. As illustrated in Figure 2f,g, as the scan rate increases, the redox current response value progressively rises, displaying a strong linear correlation between the redox peak current and the square root of the scan rate, with R^2^ values of 0.99679 and 0.98381, respectively. Similarly, a robust linear relationship is observed between the redox peak potential and logV, with R^2^ values of 0.98515 and 0.99882, respectively. This illustrates that the electrochemical process of glucose oxidation on the MXene-modified electrode is a diffusion-controlled electron transfer process [36]. Furthermore, the oxidation potential of glucose increases in direct proportion to the scan rate. The results obtained demonstrate that the paper chip exhibits excellent electrochemical stability. The position of the oxidation peak was obtained from the CV curve, the voltage value was determined, and the IT curve of the current value and the glucose concentration response were further determined. The real-time response curve of the paper chip to glucose solution was determined by the time–current method to investigate the detection effect of the paper chip on glucose testing. As shown in Figure 2j, a swift response in the current was observed upon the addition of glucose, with the concentration response ranging from 0.12 mM to 2 mM. The paper chip exhibits a high degree of sensitivity to glucose, with each drop of glucose solution eliciting a discernible current response. The current value of the response step in Figure 2j is plotted against the glucose concentration in the actual solution. A scatter plot is then generated and fitted to obtain a curve of the relationship between the current response value and the glucose concentration, as shown in Figure 2k. It is evident that in the simulated human sweat environment, affected by the glucose concentration, the current signal responds linearly.

### 3.3. Photothermal Conversion Performance of AgNP@MXene-based Paper Chips

**Figure 3:**
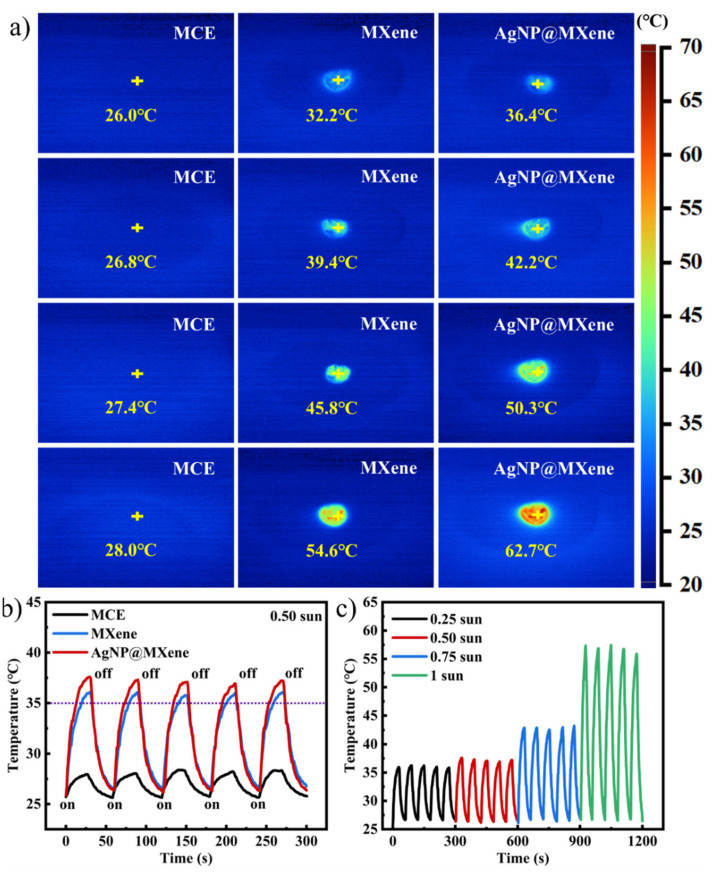
(**a**) Infrared thermal images of the B-side of MCE film, MXene, and AgNP@MXene paper chips after irradiation for 10 s with different optical powers (0.25 sun, 0.50 sun, 0.75 sun, 1 sun). (**b**) Heating–cooling cycle of the MCE membrane, MXene, and AgNP@MXene paper chip A-side. (**c**) Cyclic stability curve of AgNP@MXene paper chips.

The photothermal performance of the paper chips was assessed in a winter environment (approximately 25 °C). As illustrated in Figure 3a, infrared thermography was utilized to capture temperature images of the B-side of the MCE film, the MXene paper chip, and the AgNP@MXene paper chip. The results demonstrate a substantial increase in surface temperature of the AgNP@MXene paper chip over 10 s, reaching a maximum of 10 °C. The temperature range of 4 to 34.7 °C represents an enhancement of 4.2–8.1 °C in comparison with the MXene paper chip. The metallic nature of the Ag nano and the semi-metallic nature of MXene induce a localized surface plasmon resonance effect between them and the incident light resonance wavelength. As the direct contact with the glucose solution involved in the detection was located on the A-side of the paper chip, the irradiation time was extended. The warming curves of the MCE film, the MXene paper chip, and the A-side of the AgNP@MXene paper chip were then tested for 120 s of irradiation with different powers of the xenon lamp, as shown in S9. Under four different power irradiations, the MXene paper chip and AgNP@MXene paper chip exhibited a consistent temperature trend, with the temperature increasing continuously for 20 s and then reaching a state of equilibrium. The temperature of the AgNP@MXene paper chips was found to increase by 1.5–4.29 °C when exposed to different levels of power irradiation in comparison with MXene paper chips. The photothermal conversion efficiency was also greatly improved. Due to the higher heating rate of the AgNP@MXene paper chip, it is expected to be applied in a low-temperature environment, where it can rapidly increase the surface temperature of the object to be tested under light irradiation to reach the catalytically active temperature of the GOx interval. This would make it possible to successfully carry out glucose detection in a low-temperature environment.

The solar light intensity in the actual winter environment is approximately 0.50 sun. As illustrated in Figure 3b, the temperature response of the paper chip to light irradiation is stable, with the peak temperature being essentially the same under the same irradiation. Following cooling, the temperature rises in comparison with the initial temperature, yet the overall temperature remains unchanged. In conditions of low-level solar irradiation (0.25 sun), as illustrated in S10, the required irradiation time for the AgNP@MXene paper chip is approximately 16 s. This is approximately 9.4 s shorter than that required for the MXene paper chip when the response temperature of the paper chip A-side reaches 35 °C. In conditions of low-level solar irradiation (0.50 sun), the required irradiation time for the AgNP@MXene paper chip is approximately 6.4 s shorter than that of the MXene paper chip. When the response temperature of the paper chip’s A-side reaches 35 °C, the required irradiation time is approximately 6.4 s shorter than that of the AgNP@MXene paper chip. The MXene paper chip demonstrated a reduction in the heating time by approximately 6.1 s, suggesting that the AgNP@MXene paper chip exhibits a substantial enhancement in heating speed and detection efficiency.

To evaluate the stability of the material during the heating–cooling cycle, cyclic heating–cooling tests were performed at different irradiation powers. As demonstrated in Figure 3c, for varying degrees of irradiation, the AgNP@MXene paper chip exhibited a consistent and stable temperature rise and fall over five cycles. The peak temperature remained at a consistent level, thereby validating the paper chip’s enhanced cyclic stability in terms of its photo-thermal conversion performance.

### 3.4. Sensitivity of AgNP@MXene-based Paper Chip Sensing Under Winter Solar Insolation

**Figure 4:**
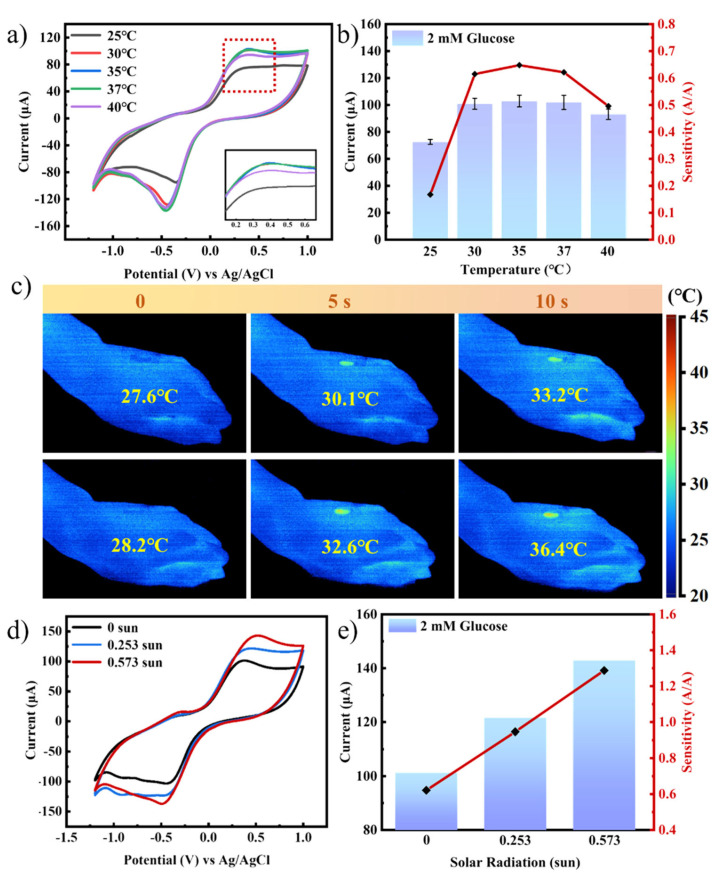
(**a**) Cyclic voltammetry curves of paper chips. (**b**) Oxidation peak current value and sensitivity of the paper chip. (**c**) Infrared thermal images of the temperature of the B-side of the paper chip after 10 s (0.253 sun, 0.573 sun). (**d**) Cyclic voltammetry curves of paper chips under noonday solar illumination. (**e**) Oxidation peak current values and detection sensitivity of paper chips under noonday solar illumination.

As with most enzymes, GOx is sensitive to temperature changes. The reaction rate of this enzyme is exponentially related to the ambient temperature at which it is located, with its optimal temperature ranging from 25 to 40 °C. Above 40 °C, the activity of GOx decreases. Figure 4a presents the cyclic voltametric curves of the AgNP@MXene paper chip in a 2 mM glucose solution at varying temperatures, with a scanning rate of 200 mV/s. As illustrated in Figure 4b, the values of oxidation peak currents are shown at varying temperatures. These correspond to the dashed boxes in Figure 4a, and their sensitivities are exhibited for the PBS background solution. It has been demonstrated that the sensitivity of the paper chip is sustained at a high level between 30 and 37 °C. Furthermore, the sensitivity of the paper chip is enhanced at 35 °C, while it is diminished at 40 °C. It is imperative to ensure that the temperature of the paper chip is maintained within the optimal range of 30–37 °C during the test to ensure the requisite high sensitivity of the paper chip.

The AgNP@MXene paper chip was then placed in the actual environment for testing. Figure 4c presents the infrared thermal images of the surface temperature of the AgNP@MXene paper chip positioned directly on the posterior of the human hand following 10 s of midday sunlight on a cloudy winter day (0.253 sun) and a sunny winter day (0.573 sun), respectively, captured by the infrared thermography camera. The photothermal conversion efficiency of AgNP@MXene is of excellent quality, enabling the paper chip to rapidly increase its temperature within 10 s. The surface temperature of the paper chip reaches 33.2 °C under cloudy winter conditions and 36.4 °C under sunny winter conditions. These temperatures are both within the optimal range for GOx. It can thus be concluded that, in the context of wearable flexible electronics, the implementation of the paper chip may facilitate the detection of glucose concentration in sweat on the skin’s surface at room temperature during winter months. This development has the potential to expand the operational scope of the paper chip.

As illustrated in Figure 4d,e, the cyclic voltametric curves of AgNP@MXene paper chips are shown in a 2 mM glucose solution, with the difference in sensitivity relative to the PBS background ambient solution under cloudy days in winter and sunny days in winter with midday solar illumination, respectively. The investigation revealed that the oxidation peak current values of AgNP@MXene paper chips under noon sunlight on cloudy days in winter and sunny days in winter were significantly higher, and the sensitivity was significantly increased, at 1.51 and 2.06 times that of the sensitivity under no light, respectively. This experimental result verifies the capability of the AgNP@MXene paper chip in the application of photothermal enhanced glucose concentration detection.

## 4. Conclusions

In this study, an AgNP@MXene hybrid was prepared using MXene as a substrate. The local surface plasmon resonance effect of Ag nanoparticles and MXene has been shown to enhance photothermal conversion efficiency under the synergistic effect of the two. The contact surface temperature on both sides of the paper chip can be significantly enhanced under light, and the temperature first rises and then tends to saturate with the prolongation of the light exposure time. It has been demonstrated that the photothermal conversion performance exhibits excellent cyclic stability, with the capacity to function multiple times without failure. The sensitivity of AgNP@MXene for glucose detection on paper chips was significantly improved by 1.51 and 2.06 times on cloudy and sunny winter days, respectively, under midday sunlight. This demonstrates the potential of AgNP@MXene paper chips for photothermal glucose concentration detection applications. It is hypothesized that the expansion of the ambient temperature range of paper chips to 25–37 °C will facilitate future applications in the domain of wearable flexible electronics.

## Data Availability

Data is available upon request.

## Supplementary Materials

The following supporting information can be downloaded at: https://www.mdpi.com/article/10.3390/s25144273/s1, Figure S1: TEM images and EDS images of AgNP@MXene at different scales: (a) 1 μm, (b) 200 nm, (c) 100 nm, (d) EDS images corresponding to Ti, C, O, F, and Ag elements in (d).; Figure S2: HRTEM images of AgNP@MXene: (a) AgNP@MXene (SAED pattern included in the inset). (b) localized magnification of the white box in (a). (c) localized magnification of the red box in (a).; Figure S3: AFM images of AgNP@MXene: (a) AFM image. (b) corresponding height contours along the line in (a).; Figure S4: Ag 3d orbital of AgNP@MXene.; Figure S5: Layered Ti_3_C_2_T_x_ XPS spectra: (a) Survey scan. (b) Ti 2p core-level spectrum. (c) C 1s core-level spectrum. (d) O 1s core-level spectrum.; Figure S6: (a) Physical photos of MXene inks. (b) FT-IR spectra of layered Ti_3_C_2_T_x_.; Figure S7: SEM images of MCE film and MXene ink writing marks on its surface: (a) low magnification SEM image of the writing marks. (b) at the boundary of the writing marks.; Figure S8: Cyclic voltammetry curves of GOx-containing and GOx-free electrodes in the solution without glucose.; Figure S9: Temperature of A-side of MCE film, MXene, and AgNP@MXene paper chip with time under different irradiations: (a) 0.25 sun, (b) 0.50 sun, (c) 0.75 sun, (d) 1 sun.; Figure S10: Heating-cooling cycle of MCE membrane, MXene, and AgNP@MXene paper chip A-side at 0.50 sun.

## References

[1] Su J., Tang L., Luo Y., Xu J., Ouyang S. (2023). Research progress on drugs for diabetes based on insulin receptor/insulin receptor substrate. Biochem. Pharmacol..

[2] Ogurtsova K., Guariguata L., Barengo N.C., Ruiz P.L.-D., Sacre J.W., Karuranga S., Sun H., Boyko E.J., Magliano D.J. (2022). IDF diabetes Atlas: Global estimates of undiagnosed diabetes in adults for 2021. Diabetes Res. Clin. Pract..

[3] Lewis D.M., Oser T.K., Wheeler B.J. (2023). Continuous glucose monitoring. BMJ.

[4] Isaacs D., Bellini N.J., Biba U., Cai A., Close K.L. (2021). Health Care Disparities in Use of Continuous Glucose Monitoring. Diabetes Technol. Ther..

[5] Maeda R., Onoue T., Mizutani K., Suzuki K., Handa T., Kobayashi T., Iwama S., Miyata T., Sugiyama M., Hagiwara D. (2025). Continuous glucose monitoring with low-glucose alerts in insulin-treated drivers with diabetes: A randomized crossover study. Diabetes. Res. Clin. Pract..

[6] Zafar H., Channa A., Jeoti V., Stojanović G.M. (2022). Comprehensive Review on Wearable Sweat-Glucose Sensors for Continuous Glucose Monitoring. Sensors.

[7] Yin J., Zhang H., Wang Y., Dong Y., Hasebe Y., Zhang Z. (2025). Noninvasive detection of glucose using electrodeposited NiCo nanoparticles on a silk derived carbon. J. Alloys Compd..

[8] Kwon H., Alù A. (2024). Active Mie-like resonance for noninvasive glucose detection. Phys. Rev. Appl..

[9] Lopes V., Abreu T., Abrantes M., Nemala S.S., De Boni F., Prato M., Alpuim P., Capasso A. (2025). Graphene-Based Glucose Sensors with an Attomolar Limit of Detection. J. Am. Chem. Soc..

[10] Sadeghi P., Noroozizadeh S., Alshawabkeh R., Sun N.X. (2025). Machine Learning-Driven D-Glucose Prediction Using a Novel Biosensor for Non-Invasive Diabetes Management. Biosensors.

[11] Padmanabhan S., Prakash J. (2025). Optimal path length identification for accurate glucose sensing with photoacoustic derived optical rotation. Opt. Lett..

[12] Liu D., Liu Z., Feng S., Gao Z., Chen R., Cai G., Bian S. (2023). Wearable Microfluidic Sweat Chip for Detection of Sweat Glucose and pH in Long-Distance Running Exercise. Biosensors.

[13] Hou Z., Gao T., Liu X., Guo W., Bai L., Wang W., Yang L., Yang H., Wei D. (2023). Dual detection of human motion and glucose in sweat with polydopamine and glucose oxidase doped self-healing nanocomposite hydrogels. Int. J. Biol. Macromol..

[14] Ali S., Abdalla I., Chen G., Xiang H., Zhu M. (2025). Non-invasive wearable nanoporous device for real-time monitoring of glucose in sweat. Compos. B. Eng..

[15] Wang Y., Zheng J., Ma Y., Li M., Zhang S., Lu Y., Wang Q., Li Y. (2025). A novel flexible non-enzymatic composite-metal glucose detection sensor in sweat based on platinum in situ plating of liquid metal. Materials. Today. Nano..

[16] Jiang S., Zhang Y., Yang Y., Huang Y., Ma G., Luo Y., Huang P., Lin J. (2019). Glucose Oxidase-Instructed Fluorescence Amplification Strategy for Intracellular Glucose Detection. ACS Appl..

[17] Ferjaoui Z., Matuszewska C., Liu J., Corvis Y., Chanéac C., Viana B., Romdhane F.B., Scherman D., Mignet N., Richard C. (2025). Highly Sensitive Detection of Glucose in the Presence of Serum Based on Signal Amplification of Persistent Luminescence Nanoparticles Functionalized by Glucose Oxidase. Adv. Opt. Mater..

[18] Franceschini F., Payo M.R., Schouteden K., Ustarroz J., Locquet J.P., Taurino I. (2023). MBE Grown Vanadium Oxide Thin Films for Enhanced Non-Enzymatic Glucose Sensing. Adv. Funct. Mater..

[19] Sigolaeva L.V., Efremova O.V., Pergushov D.V. (2023). Temperature behavior of glucose oxidase immobilized into surface-attached stimuli-sensitive copolymer microgel. Mendeleev. Commun..

[20] Ning X., Zhang Y., Yuan T., Li Q., Tian J., Guan W., Liu B., Zhang W., Xu X., Zhang Y. (2018). Enhanced Thermostability of Glucose Oxidase through Computer-Aided Molecular Design. Int. J. Mol. Sci..

[21] Naguib M., Kurtoglu M., Presser V., Lu J., Niu J., Heon M., Hultman L., Gogotsi Y., Barsoum M.W. (2011). Two-Dimensional Nanocrystals Produced by Exfoliation of Ti3AlC2. Adv. Mater..

[22] Feng A., Yu Y., Wang Y., Jiang F., Yu Y., Mi L., Song L. (2017). Two-dimensional MXene Ti3C2 produced by exfoliation of Ti3AlC2. Mat. Des..

[23] Liu Y., Xiao H., Goddard W.A. (2016). Schottky-Barrier-Free Contacts with Two-Dimensional Semiconductors by Surface-Engineered MXenes. J. Am. Chem. Soc..

[24] Wang B., Li J., Wu Z., Sheng N., Zhang M., Han Z., Jin M., Li J., Lv X., Ou K. (2022). Salinity power generation based biocompatible bacterial cellulose/MXene membrane for biological power source. Nano Energy..

[25] Bacal C.J.O., Usman K.A.S., Rashed A.O., Maina J.W., Sharp J.A., Greene G.W., Nandurkar H.H., Dwyer K.M., Razal J.M., Dumée L.F. (2024). Biocompatible MXene-reinforced molecularly imprinted membranes for simultaneous filtration and acetaminophen capture. Sep. Purif. Technol..

[26] Rafieerad A., Amiri A., Sequiera G.L., Yan W., Chen Y., Polycarpou A.A., Dhingra S. (2021). Development of Fluorine-Free Tantalum Carbide MXene Hybrid Structure as a Biocompatible Material for Supercapacitor Electrodes. Adv. Funct. Mater..

[27] Zhang C., Zong P.-a., Ge Z., Ge Y., Zhang J., Rao Y., Liu Z., Huang W. (2023). MXene-based wearable thermoelectric respiration sensor. Nano Energy.

[28] Chen R., Jia X., Zhou H., Ren S., Han D., Li S., Gao Z. (2024). Applications of MXenes in wearable sensing: Advances, challenges, and prospects. Mater. Today.

[29] Yuan R., Yang Y., Zou B., Zhang Y. (2025). MXene-enabled gas sensors for wearable breath monitoring. Chem. Eng. J..

[30] He J., Butson J.D., Gu R., Loy A.C.M., Fan Q., Qu L., Li G.K., Gu Q. (2025). MXene-Supported Single-Atom Electrocatalysts. Adv. Sci..

[31] Wang H., Li X., Deng Y., Jiang J., Ma H., Zou J. (2025). Advances in MXene-based single-atom catalysts for electrocatalytic applications. Coord. Chem. Rev..

[32] Chen H., Shao L., Ming T., Sun Z., Zhao C., Yang B., Wang J. (2010). Understanding the Photothermal Conversion Efficiency of Gold Nanocrystals. Small.

[33] Chugh V., Bhattacharjya T., Das C., Tripathy K., Bhattacharjee M. (2024). Flexible Electrochemical Sensing Label for the Detection of Glucose Adulteration in Honey. IEEE Sens. J..

[34] Zhu B., Li X., Zhou L., Su B. (2022). An Overview of Wearable and Implantable Electrochemical Glucose Sensors. Electroanalysis.

[35] Bhuvaneshwari G., Ponpandian N., Viswanathan C. (2025). Deciphering oxidation mechanism of partially oxidized Ti3C2Tₓ MXene Flakes and its electrochemical behaviour towards estradiol detection. Surf. Interfaces.

[36] Ko M., Mendecki L., Eagleton A.M., Durbin C.G., Stolz R.M., Meng Z., Mirica K.A. (2020). Employing Conductive Metal–Organic Frameworks for Voltammetric Detection of Neurochemicals. J. Am. Chem. Soc..

