# Supplementary material for: MXene-Based Flexible Paper Chip for Glucose Detection in Sweat in Low-Temperature Environments
Source: Sensors (Basel). 2025 Jul 9;25(14):4273. doi: 10.3390/s25144273 (PMC12300925; doi:10.3390/s25144273)
Supplement: Supplementary file 1 [file sensors-25-04273-s001.zip › sensors-3719494-supplementary.pdf]

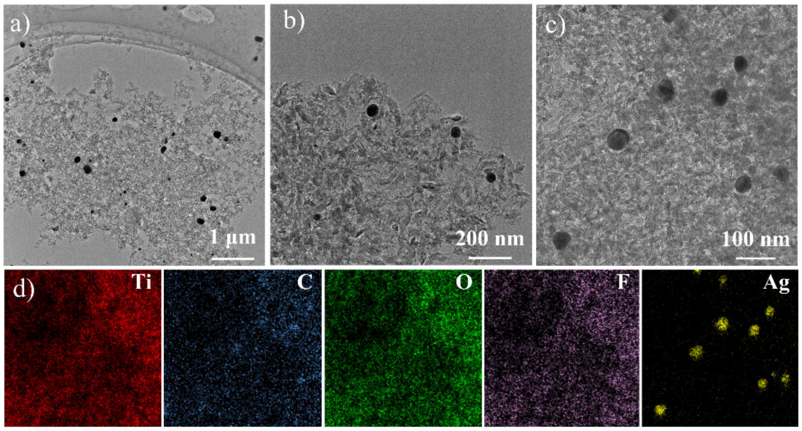

Figure S1. TEM images and EDS images of AgNP@MXene at different scales: (a) 1 μm, (b) 200 nm, and (c) 100 nm. (d) EDS images corresponding to Ti, C, O, F, and Ag elements.

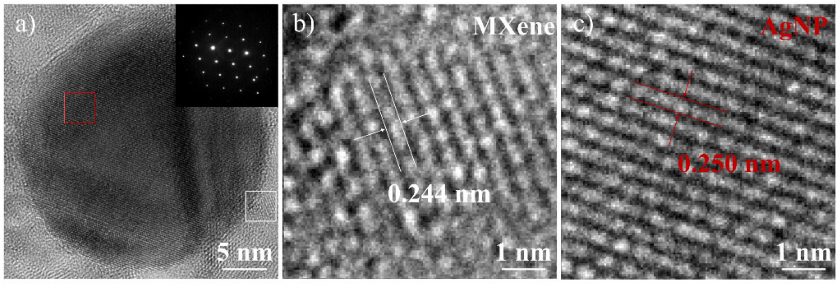

Figure S2. HRTEM images of AgNP@MXene: (a) AgNP@MXene (SAED pattern included in the inset); (b) localized magnification of the white box in (a); (c) localized magnification of the red box in (a).

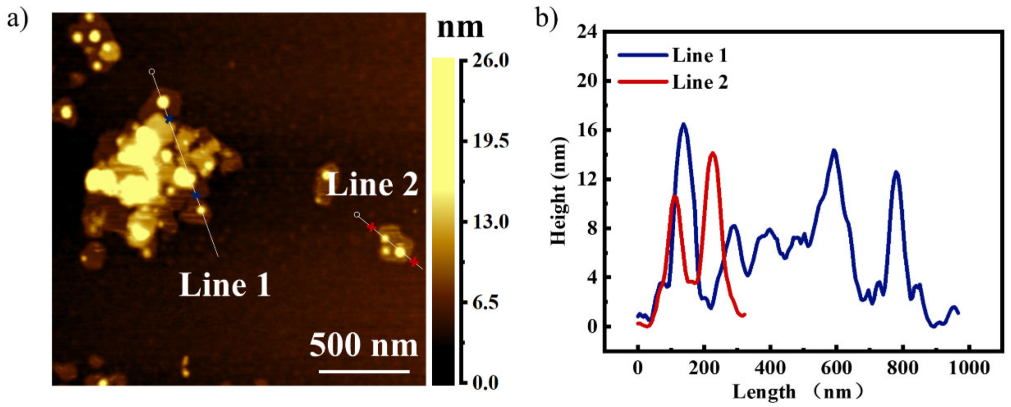

Figure S3. AFM images of AgNP@MXene: (a) AFM image; (b) corresponding height contours along the line in (a).

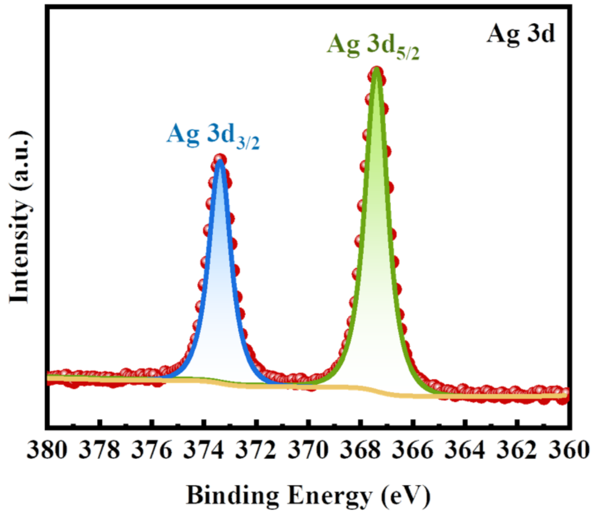

Figure S4. Ag 3d orbital of AgNP@MXene.

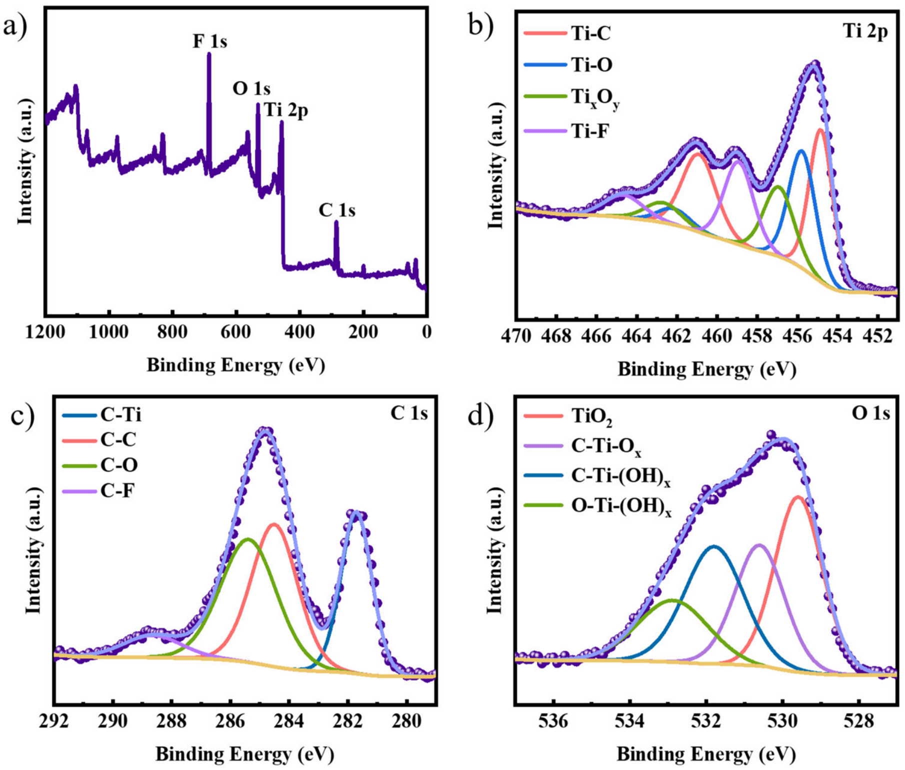

Figure S5. Layered $Ti_3C_2T_x$ XPS spectra: (a) survey scan; (b) Ti 2p core-level spectrum; (c) C 1s core-level spectrum; (d) O 1s core-level spectrum.

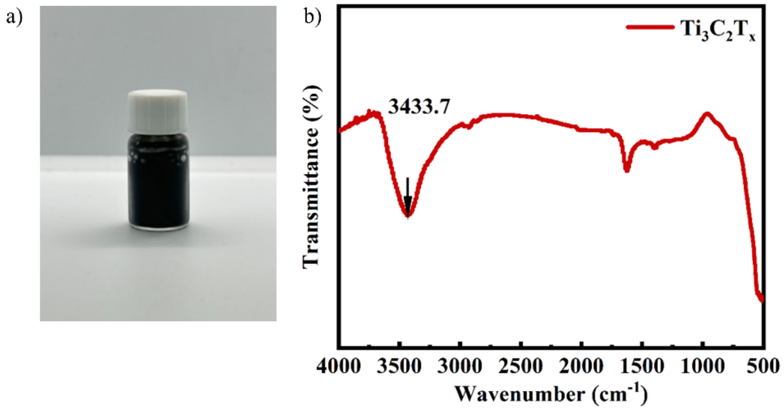

Figure S6. (a) Physical photos of MXene inks. (b) FT-IR spectra of layered $Ti_3C_2T_x$.

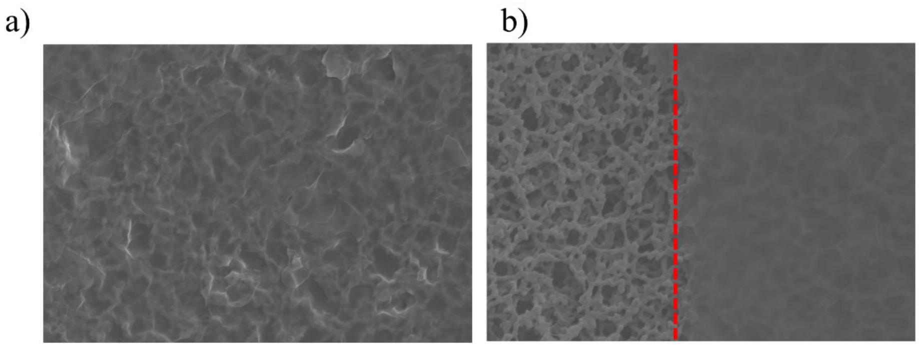

Figure S7. SEM images of MCE film and MXene ink writing marks on its surface: (a) low-magnification SEM image of the writing marks; (b) at the boundary of the writing marks.

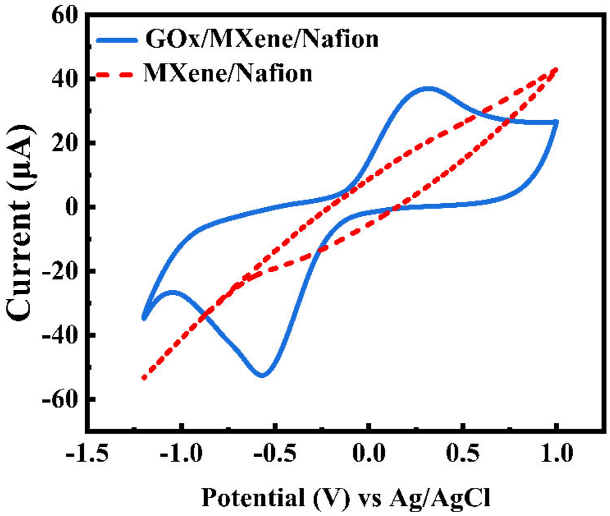

Figure S8. Cyclic voltammetry curves of GOx-containing and GOx-free electrodes in the solution without glucose.

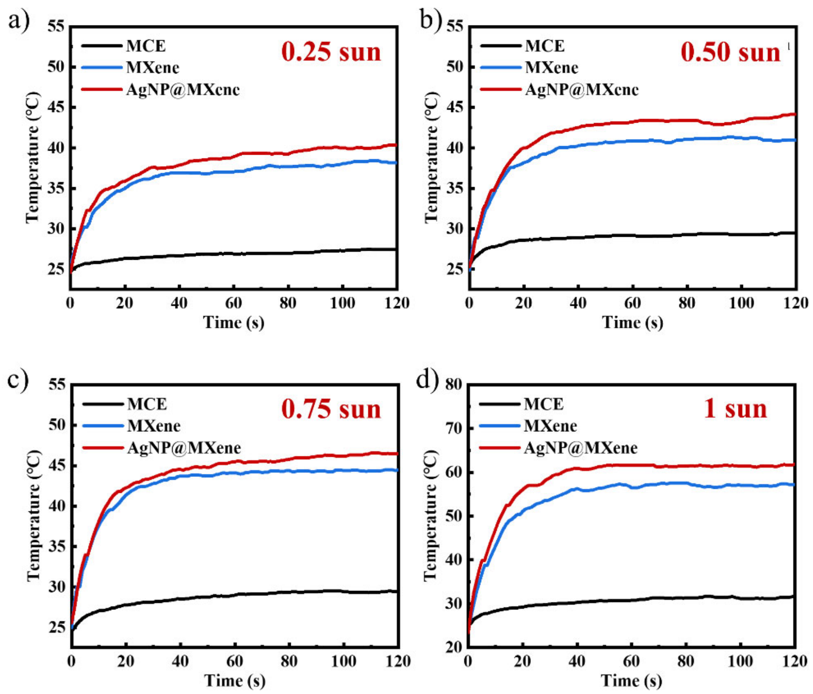

Figure S9. Temperature of A-side of MCE film, MXene, and AgNP@MXene paper chip with time under different irradiations: (a) 0.25 sun, (b) 0.50 sun, (c) 0.75 sun, and (d) 1 sun.

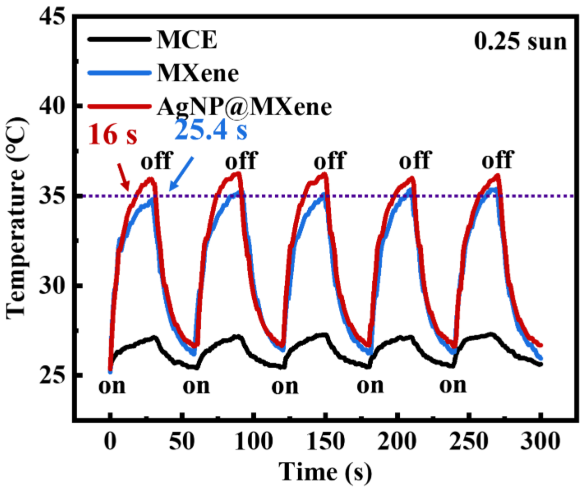

Figure S10. Heating–cooling cycle of MCE membrane, MXene, and AgNP@MXene paper chip's A-side at 0.50 sun.
